# Problematic specimens turn out to be two undescribed species of *Bignonia* (Bignoniaceae)

**DOI:** 10.3897/phytokeys.56.5423

**Published:** 2015-09-03

**Authors:** Alexandre R. Zuntini, Charlotte M. Taylor, Lúcia G. Lohmann

**Affiliations:** 1 Departamento de Botânica, Instituto de Biociências, Universidade de São Paulo, Rua do Matão, 277, 05508-090, São Paulo, SP, Brazil; 2 Missouri Botanical Garden, P.O. Box 299, St. Louis, Missouri, 63166-0299, U.S.A.

**Keywords:** Amazonia, Costa Rica, Bignoniaceae, Lianas, Neotropical Flora

## Abstract

*Bignonia* comprises 29 species of lianas characterized by eight phloem wedges, leaves usually 2-foliolate, mostly simple tendrils and opaque seed wings. The analysis of herbarium specimens in preparation for a taxonomic revision of the genus led to the recognition of two new species: (i) *Bignonia cararensis* from Costa Rica, characterized by a thyrse with lateral compound dichasia and lack of interpetiolar ridge, and (ii) *Bignonia sanctae-crucis* from Bolivia and Brazil, distinguishable by its membranous leaflets, membranous calyx and small fruits. We provide detailed descriptions, illustrations, distribution maps, initial conservation status assessments, and comparisons of the newly described taxa with closely related species.

## Introduction

*Bignonia* L. is the fifth largest genus in the Neotropical tribe Bignonieae (Bignoniaceae), with 29 species distributed from Argentina to USA (Zuntini et al. 2015). The genus comprises lianas with eight phloem wedges, leaves usually 2-foliolate, prophylls of the axillary buds foliaceous and bromeliad-like (small, decussate, triangular prophylls resembling a bromeliad), mostly simple tendrils and opaque seed wings (Lohmann and Taylor 2014). Additionally, these plants have showy rather large flowers, pink corollas, and septicidal capsules that contain numerous seeds, usually winged. Molecular studies have found that *Bignonia* is a highly supported clade that combines previously recognized genera, such as *Clytostoma* Miers ex Bureau and *Cydista* Miers (Lohmann 2006). The species of these two former genera share a variety of morphological features, such as variously cylindrical or quadrangular stems, a cupular calyx, dorso-ventrally flattened corollas, and a reduced nectariferous disk, which made their generic identification sometimes difficult. While preparing a monograph of *Bignonia* (Zuntini, Taylor and Lohmann, in prep.), more than 4,000 collections were analyzed and several problematic specimens that had been previously identified in a variety of Bignonieae genera were found to belong to *Bignonia*. However before these materials were finally identified to genus, the identity of these specimens was so unclear that they were confused with four different genera, and the flowering and fruiting specimens of each of these new species were considered to belong to different genera. Once identified as *Bignonia*, it became clear that these specimens represent two undescribed species.

These two new species are *Bignonia cararensis* Zuntini from Costa Rica and *Bignonia sanctae-crucis* Zuntini from Bolivia and western Brazil. Within *Bignonia*, these new species are not very similar and are not closely related to each other, however, these species are each similar to previously described species.

With these two new species, *Bignonia* is now composed of 31 species, with no morphological or geographical changes in the circumscription of the genus. Our results highlight the importance of large diverse herbarium collections for understanding the systematics of tropical plants, and also of broadly surveying all the specimens of a group before finalizing monographic studies rather than studying only a selected set of specimens of a given genus.

## Methods

Specimens from the following herbaria were examined: CR, F, INB, MO, NY, SPF, USJ (acronyms following Thiers 2015). The morphology descriptions follow mainly Lohmann and Taylor (2014), with additional terminology from Leaf Architecture Working Group (1999), Radford et al. (1974) and Weberling (1989). For indumentum, we follow Nogueira et al. (2013) with each trichome type described separately; peltate glandular trichomes are described according to their density as sparsely, moderately or densely lepidote, and patelliform glandular trichomes are presented here as “glands”. In the descriptions, terms inside parentheses denote rare conditions. The conservation status assessments follow IUCN guidelines (IUCN 2012), with the evaluation of geographic range based on the extent of occurrence (EOO). Distribution maps were prepared using the specimen database that was compiled as part of an ongoing monographic study of the whole genus (Zuntini, Taylor and Lohmann, in prep).

## Taxonomic treatment

### Bignonia cararensis

Taxon classificationPlantaeLamialesBignoniaceae

Zuntini sp. nov.

urn:lsid:ipni.org:names:77149637-1

#### Type.

**Figure 1.**
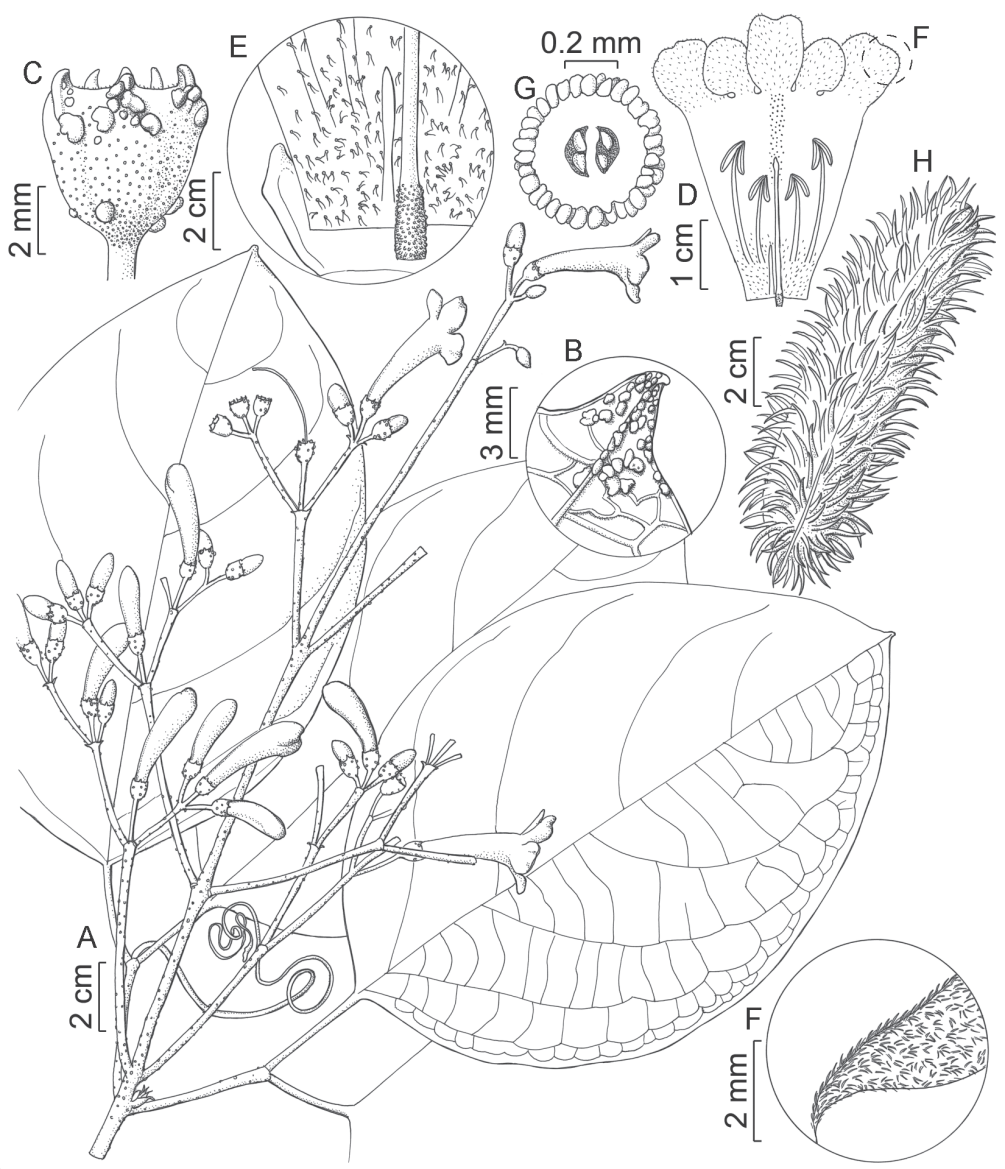
*Bignonia cararensis* Zuntini **A** Flowering branch **B** Gland cluster at leaflet apex on adaxial surface **C** Calyx **D** Opened flower **E** Detail of internal flower base, showing the glandular stipitate trichomes at corolla **F** Detail of the internal sericeous indument of the corolla lobes **G** Ovary cross-section **H** Fruit. Illustrated from *Zuñiga 90* (MO) [**A–G**] and *Weinberg s.n.* (MO-3842040) [**H**].

Costa Rica. Puntarenas: Reserva Biológica [Parque Nacional] Carara, Sector Quebrada Bonita. Sitio Area administrativa, 09°45.6'N, 084°36.0'W, 20 m, 9 February 1990, *R. Zúñiga 90* (holotype: CR-145925, mounted in two sheets!; isotypes: F!, INB!, MO!). Figure 1.

#### Diagnosis.

This new species is closely related to *Bignonia uleana* (Kraenzl.) L.G.Lohmann, but differs by the absence of interpetiolar ridges, inflorescences with compound dichasia (vs. simple dichasia in *Bignonia uleana*) and fruits up to 14 cm long with cylindrical and delicate spines (vs. longer than 16 cm with triangular and rough spines in *Bignonia uleana*). Table 1.

**Table 1.** Contrasting characters of *Bignonia cararensis* and *Bignonia uleana*.

Character	*Bignonia cararensis*	*Bignonia uleana*
Interpetiolar ridge	Absent	Present
Inflorescence lateral structure	Compound dichasia	Simple dichasia
Fruit length (cm)	11.2–14.5	16.8–26.0
Fruit spines	Cylindrical, moderately distributed	Triangular, sparsely distributed
Distribution	Costa Rica	Bolivia, Brazil and Peru

#### Description.

Lianas. **Stems** solid, cylindrical, not winged, with lenticels, without interpetiolar gland fields, without interpetiolar ridge, puberulous at least at nodes, sparsely lepidote; foliaceous prophylls caducous, cymbiform, ascending, sessile, symmetrical, 1.7–2.0 mm × ca. 1.4 mm, ciliate, sparsely lepidote, without glands; bromeliad-like prophylls present. **Leaves** 2-foliolate; petiole semi-cylindrical, 35.9–46.5 mm, without simple trichomes or puberulous, sparsely lepidote; petiolules semi-cylindrical, 25.0–45.3 mm, without simple trichomes or puberulous, sparsely lepidote; blades concolorous to slightly discolorous, chartaceous, matte, symmetrical, elliptic to widely elliptic, shortly acuminate apically, rounded basally, 17.1–23.4 × 11.0–12.8 cm, on adaxial surface puberulous at base, sparsely lepidote, with glands clustered at apex and few scattered, on abaxial surface without simple trichomes or puberulous on mid and secondary veins, sparsely lepidote, with a few scattered glands; venation pinnate, with tertiary venations mixed opposite-alternate percurrent; tendrils rarely present, simple, without simple trichomes, sparsely lepidote, with simple apex. **Inflorescences** thyrses, terminal, multi-flowered, with lateral dichasia compound and pedunculate, without simple trichomes, sparsely to moderately lepidote, primary axis ca. 255.0 mm long; bracts caducous, narrowly triangular, 1.8–2.1 × 0.5–0.6 mm, without simple trichomes, sparsely lepidote, without glands; pedicels 6.7–14.4 mm, without simple trichomes, sparsely lepidote, without glands. **Flowers** with calyx cupular, 5-toothed, sub-chartaceous, 3.7–5.9 × 4.7–6.4 mm wide at apex, ciliate, moderately lepidote, with a few scattered glands, teeth 0.6–1.4 mm; corolla purple outside, inside color unknown, infundibuliform, dorso-ventrally flattened, membranous, 40.8–75.0 mm, externally sericeous, sparsely lepidote, without glands, internally sericeous at lobes, not lepidote, with stipitate glandular trichomes at base, tube 28.4–52.5 × 2.7–3.3 mm wide at base and 10.9–14.1 mm wide at apex, lobes sub-circular, 9.7–22.0 × 10.7–15.5 mm; androecium didynamous, with stamens included, the largest 16.4–18.6 mm, the shortest 11.2–11.3 mm, without simple trichomes, not lepidote, with stipitate glandular trichomes at base, thecae 3.3–3.5 mm, staminode ca. 4.2 mm; gynoecium 25.5–27.9 mm, ovary cylindrical, verrucose, without simple trichomes, not lepidote, ovules in 2 series per locule, style not lepidote; nectariferous disk reduced. **Fruits** inflated, narrowly elliptic, 11.2–14.5 × 3.1–4.0 wide × ca. 1.4 cm thick, valves woody, without ridges, moderately echinate, without simple trichomes, not lepidote, without glands; spines cylindrical, 8.2–13.4 mm. **Seeds** unknown.

#### Distribution.

**Figure 2.**
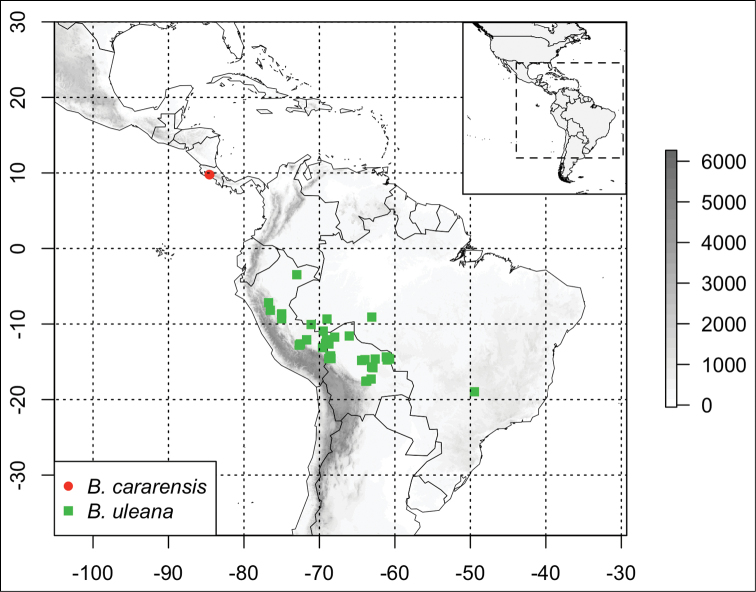
Distribution of *Bignonia cararensis* (red circles) and *Bignonia uleana* (green squares). Elevation in meters, following the scale on the right.

This species is known only from Parque Nacional Carara, in Puntarenas, Costa Rica, between 20 and 100 m elevation (Fig. 2).

#### Phenology.

Three fertile collections are documented for *Bignonia cararensis*: a single flowering specimen was collected in February and two fruiting specimens were collected in February and October.

#### Etymology.

The name is a reference to the type locality.

#### Conservation status.

The collections from the main herbaria of Costa Rica (CR, INB and USJ) were consulted, but so far this species is only documented from Parque Nacional Carara. Since *Bignonia cararensis* is known exclusively from the type locality, its full distribution cannot be accurately assessed and is here listed as Data Deficient (DD). Additional fieldwork is necessary to estimate the number of mature individuals and to assess the full extend of the species’ distribution.

#### Discussion.

This species is similar to *Bignonia uleana*, a species from Bolivia, central western Brazil and Peru. *Bignonia cararensis* can be recognized by the absence of interpetiolar ridges (vs. present in *Bignonia uleana*), the inflorescences in lateral compound dichasia (vs. lateral simple dichasia in *Bignonia uleana*), and the fruit up to 14 cm and with cylindrical delicate spines (vs. longer than 16 cm with triangular rough spines in *Bignonia uleana*) (Table 1).

The flowering collection *Zuñiga 90* was previously identified as *Cydista lilacina* A.H.Gentry [≡ *Bignonia lilacina* (A.H.Gentry) L.G.Lohmann] (Burger and Gentry 2000, Hauk 1997, *in sched*. at CR, INB and MO), and so was the sterile specimen *Acosta Vargas 826* (*in sched.* at INB and MO). *Bignonia lilacina* is similar to *Bignonia cararensis*, with which it shares cylindrical stems, large leaflets, and inflorescences in thyrses with compound lateral dichasia. However, *Bignonia cararensis* differs from *Bignonia lilacina*, an Amazonian species, by its glabrous and verrucose ovary (vs. densely lepidote and smooth in *Bignonia lilacina*), sparsely lepidote stems, inflorescences and flowers (all of these structures are densely lepidote in *Bignonia lilacina*), gland clusters borne on the adaxial surface of leaflet apices (vs. basal gland clusters on the abaxial leaflet surface in *Bignonia lilacina*) and buds with straight apices (vs. curved apices in *Bignonia lilacina*).

In contrast, the fruiting collection *Jiménez 2042* was previously identified as *Clytostoma pterocalyx* Sprague ex Urb. [≡ *Bignonia pterocalyx* (Sprague ex Urb.) L.G.Lohmann] (*in sched.* at INB), and as *Clytostoma sciuripabulum* Bureau & K.Schum. [≡ *Bignonia sciuripabulum* (Bureau & K.Schum.) L.G.Lohmann] (*in sched.* at CR and INB). The other fruiting material of this new species (*Weinberg s.n.*) was also identified as *Clytostoma sciuripabulum* (*in sched.* at MO). However, *Bignonia cararensis* differs from *Bignonia pterocalyx* by its puberulous stems and inflorescences (vs. pilose in *Bignonia pterocalyx*), 2-foliolate leaves (vs. 1-foliolate in *Bignonia pterocalyx*) and moderately echinate fruit (vs. densely echinate in B. pterocalyx). *Bignonia cararensis* differs from *Bignonia sciuripabulum* by the cylindrical stems (vs. quadrangular in *Bignonia sciuripabulum*) and apical gland clusters borne on the adaxial leaflet surface (vs. no apical clusters in *Bignonia sciuripabulum*).

*Clytostoma pterocalyx* and *Cydista lilacina* were reported as new records for Costa Rica (Burger and Gentry 2000, Hauk 1997, Jiménez and Grayum 2002) based on the specimens studied here, but with the re-identification of these specimens both of these species are now known only from South America. *Bignonia pterocalyx* is found in Venezuela and Colombia, and *Bignonia lilacina* is distributed throughout Amazonia.

The Carara National Park is located in the northern portion of the Tárcoles-Térraba floristic region, which extends through the central portion of Pacific coastal Costa Rica (Hammel et al. 2004). This region has a combination of dry and moist forests, and includes elements from Nicoya and Osa Peninsulas, where *Bignonia cararensis* might also be found.

#### Additional examined specimens.

COSTA RICA. Puntarenas: Carara Biological Reserve, 2.6 km del portón de la entrada del sendero Laguna Meandrica. Primer desviación a mano izquierda entrando, 9°48.0'N, 84°35.16'W, 100 m, 6 Apr 2000, *L.G. Acosta Vargas 826* (INB, MO); Camino a Coopecarara, 9°47.16'N, 84°36.16'W, 100 m, 11 Oct 1995, *Q. Jiménez 2042* (CR, INB); Carara Biological Reserve. 15 minute walk from entrance of Carara taking trail winding right (counter-clockwise), 9°46'N, 84°31'W, 18 Feb 1991, *R. Weinberg s.n.* (MO-3842040).

### Bignonia sanctae-crucis

Taxon classificationPlantaeLamialesBignoniaceae

Zuntini sp. nov.

urn:lsid:ipni.org:names:77149638-1

#### Type.

**Figure 3.**
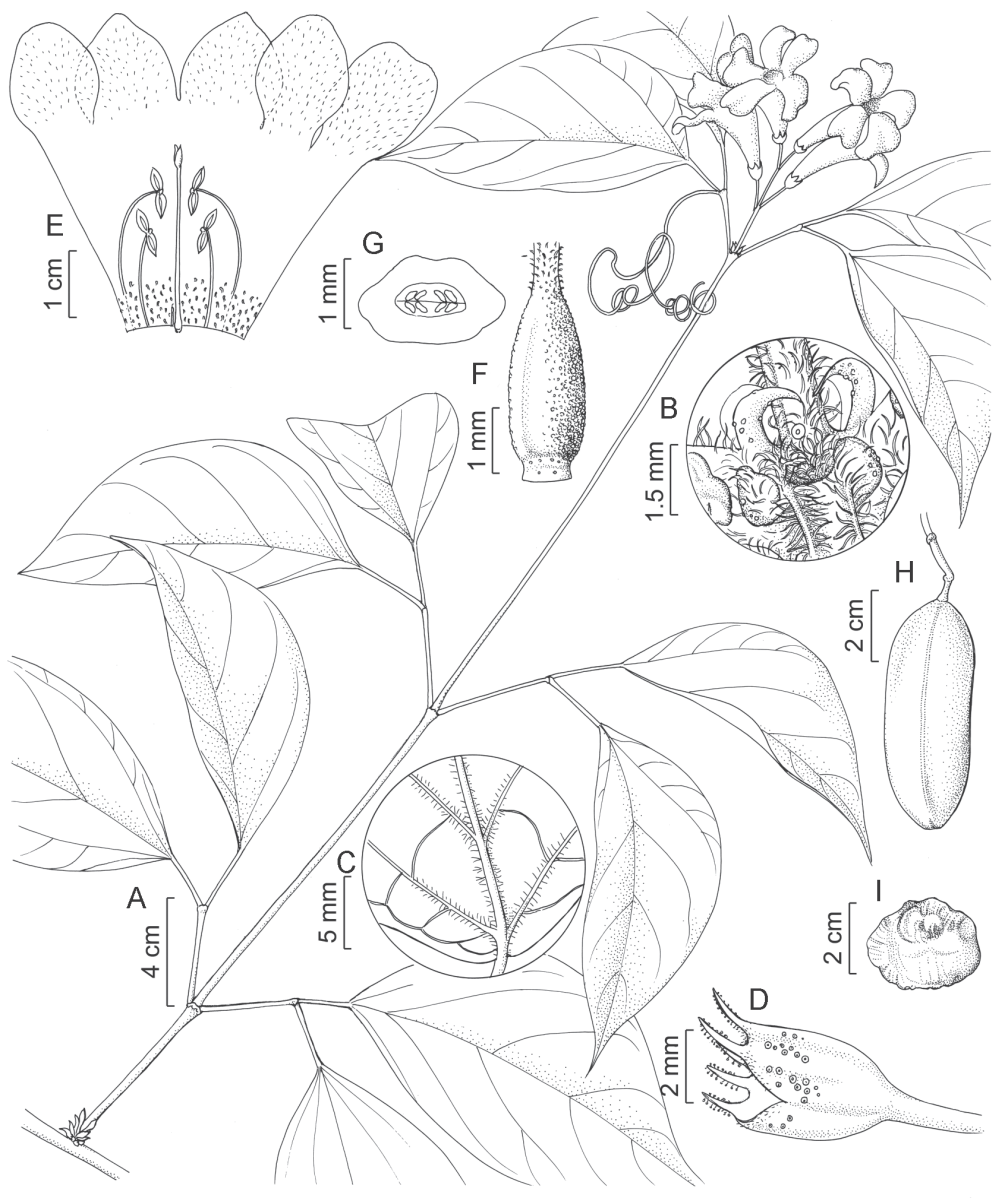
*Bignonia sanctae-crucis* Zuntini. **A** Flowering branch **B** Stem node with prophylls of the axillary buds **C** Detail of the abaxial leaflet surface **D** Calyx **E** Opened flower **F** Ovary side-view **G** Ovary cross-section **H** Fruit **I** Seed. Illustrated from *Vargas 5382* (NY) [**A,D**], *Saldias 4775* (NY) [**E–G**] and *Nee 52361* (NY) [**B, C, H, I**].

Bolivia. Santa Cruz: Prov. Ichilo. El Carmen (8 km al SSW de Buena Vista), tramo de 2km al W de la comunidad por el camino al Campamento del Río Saguayo, 17°31.98'S, 63°41.85'W, 400 m, 5 October 1996, *I.G. Vargas C. 5382 & S. Hurtado P.* (holotype: MO-5878679!; isotypes: K, NY!). Figure 3.

#### Diagnosis.

This new species is similar to *Bignonia potosina* (K.Schum. & Loes.) L.G.Lohmann, but is distinguished by its membranous leaflets with mixed opposite-alternate percurrent tertiary venation (vs. chartaceous with alternate percurrent tertiary venation in *Bignonia potosina*), membranous calyx (vs. chartaceous calyx in *Bignonia potosina*) and fruits shorter than 6.8 cm long (vs. fruits longer than 15 cm in *Bignonia potosina*). Table 2.

**Table 2.** Contrasting characters of *Bignonia decora*, *Bignonia potosina* and *Bignonia sanctae-crucis*.

Character	*Bignonia decora*	*Bignonia potosina*	*Bignonia sanctae-crucis*
Prophylls of axillary buds	Foliaceous, persistent and spreading	Falcate, caducous and ascending	Falcate, caducous and ascending
Leaf texture	Sub-chartaceous to chartaceous	Chartaceous to sub-coriaceous	Membranous
Leaf tertiary venation	Alternate percurrent	Alternate percurrent	Mixed opposite-alternate percurrent
Inflorescence	Thyrse	Raceme	Raceme
Calyx texture	Chartaceous	Chartaceous	Membranous
Fruit length (cm)	14.7–37.7	12.0–24.0	Ca. 6.8
Distribution	Bolivia, Brazil, Ecuador and Peru	Central America	Bolivia and Brazil

#### Description.

Lianas. **Stems** solid, tetragonal, winged or ribbed, with lenticels, without interpetiolar gland field, with interpetiolar ridge, puberulous to pilose at least at nodes, sparsely lepidote; foliaceous prophylls caducous, falcate (subulate), ascending, stipitate, asymmetrical, 0.9–2.3 mm × 0.5–1.5 mm, without simple trichomes, sparsely lepidote, with a few glands on abaxial surface (no glands); bromeliad-like prophylls present. **Leaves** 2-foliolate; petiole semi-cylindrical, (6.1–)15.1–38.9 mm, pubescent, puberulous or pilose, sparsely lepidote; petiolules semi-cylindrical, 9.7–29.8 mm, pilose, sparsely lepidote; blades slightly discolorous, membranous, matte, slightly asymmetrical to asymmetrical, elliptic to widely ovate, acuminate to long acuminate apically, rounded basally (short attenuate), 8.3–13.3(–18.9) × 5.1–8.5(–12.3) cm, on adaxial surface without simple trichomes, densely lepidote, without glands, on abaxial surface pilose along midvein and secondary veins, sparsely lepidote, with a few scattered glands; venation pinnate, with tertiary venations mixed opposite-alternate percurrent; tendrils rarely present, simple, without simple trichomes, sparsely lepidote, with simple apex. **Inflorescences** racemes, terminal, 2–4-flowered, without simple trichomes or puberulent, sparsely lepidote, primary axis 8.3–13.8 mm long; bracts caducous, not observed; pedicels 5.5–10.0 mm, without simple trichomes, moderately lepidote. **Flowers** with calyx cupular, 5-toothed, membranous, 4.5–6.0 × 4.3–5.1 mm wide at apex, ciliate, moderately lepidote, with glands clustered in columns, teeth 0.6–1.5 mm; corolla creamish outside, yellowish inside, infundibuliform, dorso-ventrally flattened, membranous, 30.6–52.8 mm, externally pubescent at lobes, moderately lepidote, without glands, internally with pubescent lobes, not lepidote, with shortly stipitate glandular trichomes at base, tube 19.8–40.0 × 2.1–5.0 mm wide at base and 9.2–16.3 mm wide at apex, lobes rounded or oblong, 9.7–15.4 × 8.8–15.2 mm; androecium didynamous, with stamens included, the largest 10.7–18.0 mm, the shortest 6.4–11.7 mm, without simple trichomes, not lepidote, with shortly stipitate glandular trichomes at base, thecae 1.7–3.6 mm, staminode 0.9–2.5 mm; gynoecium 21.5–29.2 mm, ovary ovoid to cylindrical, smooth, without simple trichomes (pilose at apex), densely lepidote, ovules in 4 series per locule, style sparsely lepidote at base; nectariferous disk reduced. **Fruits** inflated, oblong, ca. 6.8 × 2.8 wide × 0.8 cm thick, valves woody, without ridges, smooth, without simple trichomes, sparsely lepidote, without glands. **Seeds** beige, thin, transversally elliptic to narrowly transversally oblong, symmetrical, 13.7–24.3 × 28.5–39.4 mm, with two opaque wings; seed body flattened, 0.8–1.2 mm thick.

#### Distribution.

**Figure 4.**
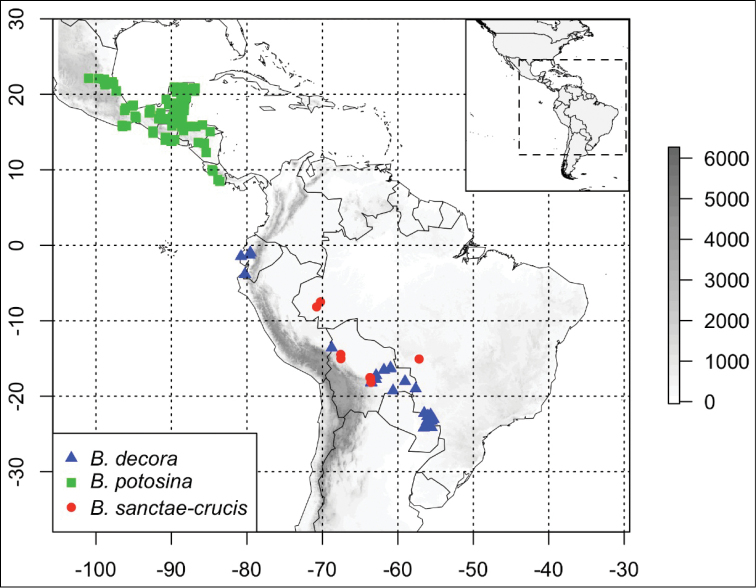
Distribution of *Bignonia sanctae-crucis* (red circles), *Bignonia decora* (blue triangles), and *Bignonia potosina* (green squares). Elevation in meters, following the scale on the right.

This species is found in evergreen or semideciduous forests in Western Amazonia, occurring in Bolivia (Beni, La Paz and Santa Cruz) and Brazil (Acre, Amazonas and Mato Grosso), between 160 to 700 m alt. (Fig. 4).

#### Phenology.

This species was collected with flowers in June, September, October and November. A single fruiting specimen was collected in July.

#### Conservation status.

*Bignonia sanctae-crucis* is known from only seven locations but is considered Least Concern (LC) given its wide extent of occurrence (over 600.000 km^2^) and the different physiognomies where it occurs, including secondary formations. The number of locations where this species is known to occur is likely underestimated because *Bignonia* species are usually not densely distributed and because this entire region is not well documented floristically. Additional fieldwork is needed in order to fully document the extent of distribution of this species.

#### Etymology.

The epithet refers to the type locality, the Department of Santa Cruz (Bolivia), where most specimens were collected.

#### Discussion.

*Bignonia sanctae-crucis* and *Bignonia potosina* share quadrangular and ribbed (winged) stems, prominent interpetiolar ridges, falcate and caducous prophylls, and few-flowered racemes. Apart from being morphologically similar, these species are also closely related and can be confused. However, *Bignonia sanctae-crucis* can be distinguished from *Bignonia potosina* by the membranous calyx (vs. chartaceous in *Bignonia potosina*) and fruits shorter than 6.8 cm long (vs. fruits longer than 15 cm in *Bignonia potosina*) (Table 2). These two species are also geographically widely separated, with *Bignonia sanctae-crucis* found in Bolivia and central to western Brazil while *Bignonia potosina* is widely found in Mexico and Central America but not in South America. *Bignonia sanctae-crucis* can also be confused with the sympatric species *Bignonia decora* (S.Moore) L.G.Lohmann due to the quadrangular stems shared by both species. However, *Bignonia sanctae-crucis* can be recognized by its falcate and caducous prophylls (vs. foliaceous and persistent in *Bignonia decora*), few-flowered racemes (vs. multi-flowered thyrses in *Bignonia decora*) and fruit without ridges (vs. three longitudinal ridges in *Bignonia decora*) (Table 2). Quadrangular stems are also characteristic of *Bignonia sciuripabulum* (Bureau & K.Schum.) L.G.Lohmann, a distantly related species (Zuntini and Lohmann, in prep.) that has a verrucose and glabrous ovary (vs. smooth and lepidote in *Bignonia sanctae-crucis*) and echinate fruits (vs. smooth in *Bignonia sanctae-crucis*); *Bignonia sciuripabulum* is found in Amazonia and the Atlantic forest of Brazil.

The only fruiting material of this new species (*Nee 52361*) was previously identified as Cydista cf. decora (S.Moore) A.H.Gentry [≡ *Bignonia decora*] (*in sched.* at NY), a closely related species. The flowering specimens of *Bignonia sanctae-crucis*, however, were identified as *Clytostoma sciuripabulum* Bureau & K.Schum. [≡ *Bignonia sciuripabulum*], *Clytostoma uleanum* Kraenzl. [≡ *Bignonia uleana*], and some other *Clytostoma* species (*in sched.* at MO and NY). The thin-textured corolla probably confused the generic identification, given that most *Cydista*, as previously circumscribed, were characterized by thicker corollas whereas such thin corollas were characteristic of the previously recognized *Clytostoma*. Despite its corolla texture, *Bignonia sanctae-crucis* is not closely related to the species that were included in *Clytostoma*, and does not have the verrucose glabrous ovary that is characteristic of that group.

#### Additional examined specimens.

BOLIVIA. Beni: Rurrenabaque, Rurrenabaque, 14°28'S, 67°34'W, 333 m, 8 Oct 1921, *White 874* (NY). La Paz: Alto Beni, Concesión de San Jose de Papay, 15°02'S, 67°33'W, 500 m, 23 Oct 1987, *E. Vargas 2022* (LPB, MO); San Buena Ventura, 500 m, 29 Nov 1901, *R.S. Williams 363* (NY). Santa Cruz: Cercado, Lomas del Rio Cúcha, 450 m, 28 Oct 1925, *J. Steinbach G. 7307* (F, MO); Ibáñez, Gorge of Río Bermejo, 6.5km (by road) W of the checkpoint at Angostura, 18°10'S, 63°33'W, 690 m, 25 Jul 2003, *M.H. Nee 52361* (LPB, NY, USZ). Ichilo, 2 km W of Center of San Carlos, older secondary growth along highway from Buena Vista to Villa Tunari, 17°24.5'S, 63°45'W, 310 m, 31 Oct 1999, *M.H. Nee 50398* (NY); Ichilo, Estáncia San Rafaél (propiedad de la Unversidad NUR), 16 km SW de Buena Vista, 17°36'S, 63°36'W, 432 m, 1 Oct 1996, *M. Saldias P. 4775* (NY, USZ). BRAZIL. Acre: Tarauacá, 1–3 km east of Rio Tarauacá, 24 Sep 1968, *G.T. Prance 7513* (K, INPA, MG, MO, NY). Amazonas: Envira, Rio Juruá, Basin of Rio Jurua, near mouth of Rio Embira, 7°30'S, 70°15'W, 160 m, 28 Jun 1933, *B.A. Krukoff 5046* (MICH, MO, NY, US). Mato Grosso: Barra do Bugres, Fazenda Ochsenfeld, 23 Oct 1995, *G. Hatschbach 63777* (MBM, SPF).

## Supplementary Material

XML Treatment for Bignonia cararensis

XML Treatment for Bignonia sanctae-crucis

## References

[B1] Burger W Gentry AH (2000) Bignoniaceae. In: Burger W (Ed.) Flora costaricensis. Fieldiana, Bot, n.s., 41: 77–162.

[B2] Hammel BE Grayum MH Herrera C Zamora N (2004) 1 Manual de plantas de Costa Rica. Missouri Botanical Garden Press, St. Louis, 324 pp.

[B3] Hauk WD (1997) A review of the genus *Cydista* (Bignoniaceae). Annals of the Missouri Botanical Garden 84: 815–840. doi: 10.2307/2992028

[B4] IUCN (2012) IUCN red list categories and criteria: Version 3.1. Second edition. IUCN Red List Unit, Cambridge, 32 pp.

[B5] Jiménez MQ Grayum MH (2002) Vegetación del Parque Nacional Carara, Costa Rica. Brenesia 57–58: 25–66.

[B6] Leaf Architecture Working Group (1999) Manual of leaf architecture: morphological description and categorization of dicotyledons and net-veined monocotyledonous angiosperms. Smithsonian Institution, Washington, 65 pp.

[B7] Lohmann LG (2006) Untangling the phylogeny of neotropical lianas (Bignonieae, Bignoniaceae). American Journal of Botany 93: 304–318. doi: 10.3732/ajb.93.2.304 21646191 10.3732/ajb.93.2.304

[B8] Lohmann LG Taylor CM (2014) A new generic classification of Tribe Bignonieae (Bignoniaceae). Annals of the Missouri Botanical Garden 99: 348–489. doi: 10.3417/2003187

[B9] Nogueira A Ottra JHL El Guimarães E Machado SR Lohmann LG (2013) Trichome structure and evolution in Neotropical lianas. Annals of Botany 112: 1331–1350. doi: 10.1093/aob/mct201 24081281 10.1093/aob/mct201 PMC3806532

[B10] Radford AE Dickison WC Massey JR Bell CR (1974) 1 Vascular Plant Systematics. HarperCollins, New York, 891 pp.

[B11] Thiers B (2015) Index Herbariorum: A global directory of public herbaria and associated staff. New York Botanical Garden’s Virtual Herbarium. http://sweetgum.nybg.org/ih/

[B12] Weberling F (1989) 1 Morphology of flowers and inflorescences. Cambridge University Press, Cambridge, UK, 415 pp.

[B13] Zuntini AR Taylor CM Lohmann LG (2015) Deciphering the Neotropical *Bignonia binata* species complex (Bignoniaceae). Phytotaxa 219: 69–77. doi: 10.11646/phytotaxa.219.1.5

